# Comparison of Supported Ionic Liquid Membranes and Polymeric Ultrafiltration and Nanofiltration Membranes for Separation of Lignin and Monosaccharides

**DOI:** 10.3390/membranes10020029

**Published:** 2020-02-14

**Authors:** Ricardo Abejón, Javier Rabadán, Aurora Garea, Angel Irabien

**Affiliations:** Chemical and Biomolecular Engineering Department, University of Cantabria. Avda. Los Castros s/n, 39005 Santander, Cantabria, Spain; javier.rabadan.mtz@gmail.com (J.R.); gareaa@unican.es (A.G.); irabienj@unican.es (A.I.)

**Keywords:** supported ionic liquid membranes, separation, imidazolium-based ionic liquid, ultrafiltration and nanofiltration membranes, separation factors, Kraft lignin, lignosulphonate, monosaccharides

## Abstract

Lignin is one of the three main components of lignocellulosic biomass and must be considered a raw material with attractive applications from an economic and ecological point of view. Therefore, biorefineries must have in mind the most adequate processing to obtain high-quality lignin and the separation tasks that play a key role to improve the purity of the lignin. Separation techniques based on membranes are a promising way to achieve these requirements. In this work, the separation performance of the SILM (Supported Ionic Liquid Membrane) formed with [BMIM][DBP] as IL (Ionic Liquid) and PTFE as membrane support was compared to a nanofiltration (NF) membrane (NP010 by Microdyn-Nadir) and two ultrafiltration (UF) membranes (UF5 and UF10 by Trisep). The SILM showed selective transport of Kraft lignin, lignosulphonate, xylose, and glucose in aqueous solutions. Although it was stable under different conditions and its performance was improved by the integration of agitation, it was not competitive when compared to NF and UF membranes, although the latter ones suffered fouling. The NF membrane was the best alternative for the separation of lignosulphonates from monosaccharides (separation factors around 75 while SILM attained only values lower than 3), while the UF5 membrane should be selected to separate Kraft lignin and monosaccharides (separation factors around 100 while SILM attained only values below 3).

## 1. Introduction

The sustainable management of forest resources is a great opportunity to avoid deforestation and forest degradation while obtaining direct benefits for people in those rural areas, which complement additional benefits for the environment. Sustainably exploited forests must provide the balance between biodiversity preservation and income generation through forest products and services [1,2]. Among these forest products, wood must be highlighted. On the one hand, wood construction is an increasing global phenomenon as both the result of more environmentally friendly policies and the technical needs to increase construction productivity [3]. Therefore, mechanical transformation products derived from wood are called to replace non-renewable materials in the building industry and furniture manufacture [4]. On the other hand, the chemical transformation of lignocellulosic raw materials produces a large variety of renewable chemical compounds, which could be directly used as chemicals or considered as building blocks for the synthesis of other valuable substances [5].

Traditional wood chemical transformation has been led by the pulp and paper industry, which was totally focused on the optimal use of cellulose. However, the other two main components of lignocellulosic biomass (hemicellulose and lignin) were not taken into consideration during the optimization of these transformation processes. Nowadays, biorefineries propose a new model for the integral use of lignocellulosic biomass. The global objective of an effective lignocellulosic biorefinery must be to maximize the added value generated from the entire biomass feedstock, including the hemicellulose and lignin fractions [6]. In fact, the economic viability of a lignocellulosic biorefinery depends on the way how hemicellulose and lignin present in biomass are valorized. Several different options exist for the valorization of hemicellulose and lignin [7,8,9,10,11], but all of them require adequate biomass pretreatments. Due to the recalcitrant nature of lignocellulosic biomass, simple direct treatments are unviable, and it must be fractionated to separate lignin (an aromatic polymer) from cellulose and hemicellulose (carbohydrate polymers).

Focusing on lignin valorization, this component must be considered the main renewable raw material for aromatic chemicals. The unselective depolymerization of lignin under aggressive conditions can result in a mixture of benzene, toluene, xylene and phenol. In contrast, under milder conditions, a high variety of products (depending on the specific lignin structure and blocks) can be obtained and most of these products fall outside traditional petrochemical routes [12]. To take the best from lignin, the lignocellulosic biomass fractionation must be reconfigured to avoid the production of only low-purity lignin with low recovery yield. Therefore, new processes must be proposed for lignin separation and purification.

Separation processes based on membranes can play a relevant role in this new scenario, since they have demonstrated high usefulness in current biorefinery applications [13]. Lignin processing is not an exception and pioneer examples of application of ultrafiltration (UF) membranes to the treatment spent Kraft and bisulphite liquors were published in the 1980s [14,15,16]. After these initial works, the employment of ceramic UF membranes for the recovery of Kraft lignin from spent liquors without pH or temperature changes was deeply investigated during the first decade of this century [17,18,19], and it was extended to further applications, such as the separation of lignin-derived chemicals like vanillin [20]. In addition to ceramic ones, polymeric UF membranes have been tested as well in the treatment of spent Kraft and sulphite liquors [21,22,23]. The development of new lignocellulosic biomass pretreatment processes, such as organosolv and alkaline oxidation fractionation, has provided new opportunities for the implementation of nanofiltration (NF) membranes for lignin processing [24,25,26,27,28,29]. The use of membrane cascades, which can combine high selectivity and yield [30,31], has been also proposed for lignin recovery and purification [32,33]. pointing out some review papers for further information about the use of UF and NF membranes for lignin processing [34,35,36].

Apart from pressure-assisted membrane technologies, liquid membranes have been applied to the separation of lignin. A liquid membrane is just a liquid barrier that separates two liquid phases of different composition and allows the transport of at least a solute between them [37]. For the separation of lignosulphonates, the proposed liquid membranes were formulated with organic amines as carriers for facilitated transport [38], such as trioctylamine or trilaurylamine, dissolved in dichloroethane, 1-decanol or sunflower oil [39,40,41,42,43,44,45]. In the case of Kraft lignin, Aliquat 336 dissolved in kerosene was mentioned as an effective liquid membrane [46]. However, ionic liquids (ILs) can be ideal candidates for the formulation of new liquid membranes to be used in lignin separation. Several researchers have investigated the potential of ILs to dissolve, separate and purify lignin [47,48,49,50,51,52,53,54].

Previous works of this research group tested several supported ionic liquid membranes (SILMs) and allowed the identification of the system composed by polytetrafluoroethylene (PTFE) as membrane support and 1-butyl-3-methylimidazolium dibutylphosphate ([BMIM][DBP]) as IL as a valid option for selective transport of lignin and monosaccharides [55,56]. The main objective of this work was the analysis of the technical applicability of the previously identified selective SILM for the separation of lignin (Kraft lignin and lignosulphonate) and monosaccharides. This analysis included the stability of the SILM and the study of the influence of the SILM preparation and operation conditions. This way, once the separation process based on the SILM was characterized, it was compared to the process that employed polymeric ultrafiltration and nanofiltration membranes for the same purpose in order to study the competitiveness of both technologies.

## 2. Experimental

### 2.1. Materials and Methods

An imidazolium-based IL, 1-butyl-3-methylimidazolium dibutylphosphate ([BMIM][DBP]), was used as received from Iolitec. Sodium lignosulphonate was purchased from TCI Chemicals and alkali Kraft lignin (low sulfonate content), D-(+)-glucose (>99.5%) and D-(+)-xylose (>99%) were provided by Sigma-Aldrich. Polytetrafluoroethylene (PTFE) disc filters (47 mm diameter and 0.45 µm pore) from Filter-Lab were employed as membrane supports for the SILM. A polymeric NF membrane (Mycrodyn-Nadir) and two UF membranes (Trisep UF5 and UF10), all of them made of polyethersulfone (PES) with polypropylene (PP) support layer, were employed in the tests as 47 mm discs. Their main characteristics provided by the corresponding manufacturers are compiled in Table 1.

Lignin and monosaccharides concentrations were determined by a UV-VIS spectrophotometer DR 5000, using a wavelength of 280 nm for the lignin [57] and of 575 nm for monosaccharides, according to the dinitrosalicylic acid method for determination of reducing sugars [58]. Triplicate concentration measures were performed with 1 min intervals among them.

### 2.2. SILMs Preparation y Experimental Tests

The SILMs were prepared using PTFE membranes as supports and [BMIM][DBP] as IL. Firstly, the membrane and the IL were introduced in a vacuum oven (<35 mbar) in separated Petri disks to eliminate the water, gases and any other traces of volatile compounds. Later, the membrane was soaked in the IL, keeping the vacuum for 24 h to favor the proper impregnation by removal of air from the membrane pores. Lastly, the liquid excess over the membrane surface was removed by allowing to drip overnight. This way, the membrane was ready to be placed in the membrane cell.

The SILM experimental tests were carried out in a membrane cell designed for this specific project, previously employed for the characterization of several SILMs [55]. The cell is composed of two identical compartments (volume 120 mL) separated by the SILM. Two different types of experiments were performed. On the one hand, the feed and stripping solutions were poured into the cell at the same time and the compartments were closed. On the other hand, the feed and stripping solutions were poured into the cell at the same time and two tubes were introduced in the feed compartment to allow inlet and outlet streams. These tubes were connected to an external reservoir under continuous agitation. The flowrate of these streams was 15 mL/s, so a quick replacement of the fluid in the cell compartment by the fluid from the reservoir was assured. In both cases, samples were taken at regular time intervals from both compartments. All the experiments were carried out at room temperature.

### 2.3. UF and NF Membranes Experimental Tests

A lab-scale, dead-end HP4750 stirred cell from Sterlitech was purchased for UF and NF experiments. The membrane cell can accommodate any 47 to 50 mm diameter disc membranes, resulting 14.6 cm^2^ of effective membrane area. The filtration cell was filled first with 300 mL of ultrapure water for the assessment of membrane permeability and then with the corresponding lignin or monosaccharide solution for characterization of the separation performance of the membranes. The membrane cell was pressurized by a N_2_ cylinder, and the different applied pressures were adjusted by the control valve of the cylinder. The permeate flux was calculated with a laboratory balance and a chronometer. Samples for determination of the lignin and monosaccharide concentrations in the feed, permeate and retentate solutions were taken. Triplicate flux and concentration measures were performed with 3 min intervals among them.

### 2.4. Transport Model

The flux *J* of a solute that is transported through a SILM is proportional to the gradient of the solute concentration *C* between both solutions:

(1) J=k·ΔC

where *k* is the proportionality constant that can be defined as the permeability. To model the evolution of the concentration of the solute in the feed compartment *C_F_* during the closed configuration of the cell (without inlet and outlet streams), the corresponding mass balance to the feed compartment must be applied:

(2) $$V\cdot \frac{dC_{F}}{dt}=-k\cdot \Delta C\cdot A$$

where *V* is the volume of the feed solution and *A* the active surface of the SILM. From this formulation, the classical equation that defines the evolution of the solute concentration in the feed compartment *C_F_* (and in an equivalent way the concentration in the stripping compartment *C_S_*) of the SILM cell as a function of the effective transport parameter *K* can be obtained [59]:

(3) $$C_{F}=\frac{C_{0}}{2}\cdot \left(1+e^{-K\cdot t}\right)$$

(4) $$C_{S}=\frac{C_{0}}{2}\cdot \left(1-e^{-K\cdot t}\right)$$

(5) $$K=\frac{2\cdot k\cdot A}{V}$$

For the case of the cell under open configuration (inlet and outlet streams in the feed compartment), the system formed by the feed compartment, the external agitated reservoir and the connection tubes can be considered as an ideally stirred unit with *V_F_* volume. Within these conditions, the evolution of the concentrations was defined by the following equations:

(6) $$C_{F}=\frac{C_{0}}{1+\gamma }\cdot \left(\gamma +e^{-K_{F}\cdot t}\right)$$

(7) $$C_{S}=\frac{C_{0}}{1+\gamma }\cdot \gamma \left(1-e^{-K_{S}\cdot t}\right)$$

(8) $$K_{F}=\frac{k\cdot A}{V_{F}}$$

(9) $$K_{S}=\frac{k\cdot A}{V_{S}}$$

(10) $$\gamma =\frac{V_{F}}{V_{S}}$$

where *K_F_* and *K_S_* are the effective transport parameter of the feed and stripping sides respectively and *γ* the relation between both volumes.

The performance of the UF and NF membranes was characterized by the determination of the corresponding permeate fluxes and solute rejections. On the one hand, the permeate fluxes *J_P_* were defined as functions of the membrane permeability *L_P_*, the applied pressure Δ*P* and the baseline flow *J*_0_ (the baseline flow was only considered for the UF10 membrane, since the other two membranes did not allow the permeation until pressure was applied):

(11) $$J_{P}=L_{P}\cdot \Delta P+J_{0}$$

On the other hand, the solute rejections *R* were defined by the equation:

(12) $$R=100\frac{C_{IN}-C_{P}}{C_{IN}}$$

where *C_IN_* and *C_P_* represent the solute concentrations measured in the feed and permeate streams of the membrane cell, respectively.

## 3. Results and Discussion

**Figure 1:**
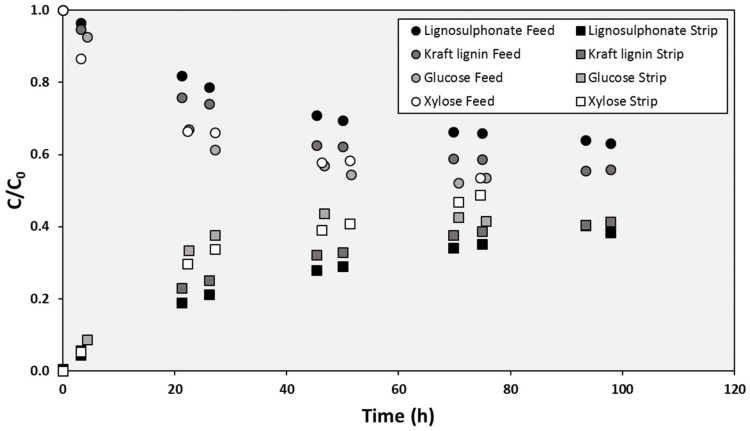
Evolution of the concentration of the different solutes in the cell compartments during a typical SILM test.

The prepared SILM, with [BMIM][DBP] supported in PTFE membrane, had demonstrated selective transport of different lignin types and monosaccharides. The evolution of the concentrations in the feed and stripping compartments when single solutions of the four selected solutes (lignosulphonate, Kraft lignin, glucose and xylose) were employed was very different. As shown in Figure 1, the transport of the monosaccharides was faster than the transport of the lignins: While 70 h was time enough to achieve monosaccharides concentrations that could be considered equilibrium values, the normalized concentrations of lignins in the stripping compartments were still below 0.4 after more than 100 h.

Two different aspects that could affect the stability of the prepared SILM were investigated: on the one hand, related to the SILM preparation procedure, the temperature in the vacuum oven during the humidity removal and IL impregnation was reduced from 70 °C (standard temperature in the procedure) to 30 °C. On the other hand, related to the cell operation, fluid replacement by an open configuration (15 mL/s inlet and outlet streams with an external agitated reservoir of 470 mL) was implemented.

**Figure 2:**
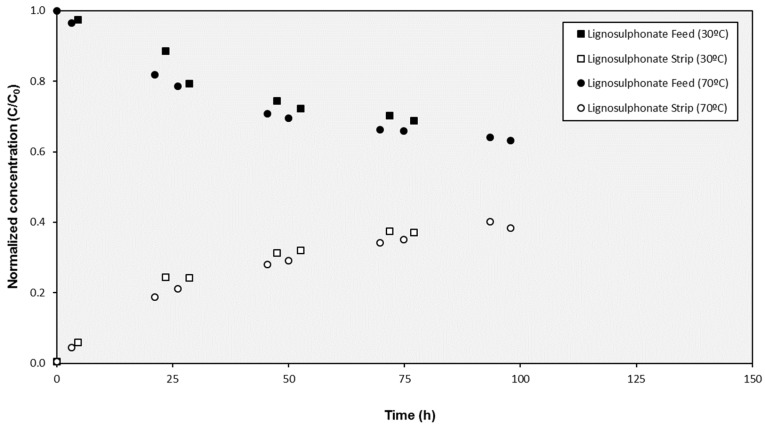
Evolution of the concentration of lignosulphonates in the cell compartments during tests with SILMs prepared at different temperatures.

As can be observed in Figure 2, the results obtained with the SILMs prepared at different temperatures were equivalent and no influence on the SILM performance could be identified (the values of the effective transport parameters K were 0.0156 and 0.0162 h^−1^ for the SILMs prepared at 30 °C and 70 °C respectively).

**Figure 3:**
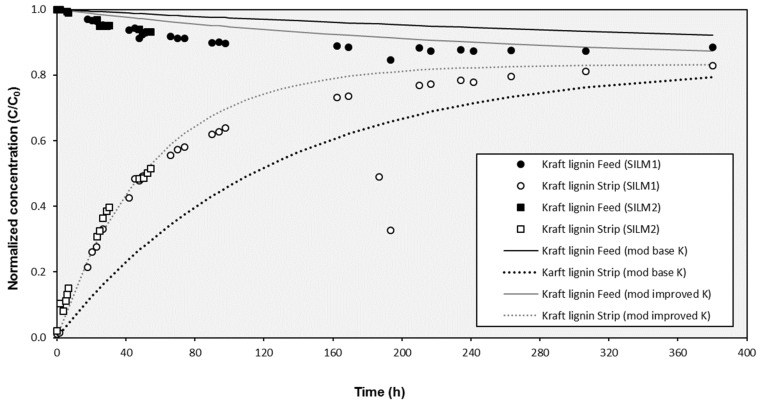
Evolution of the experimental concentration of Kraft lignin in the cell compartments during the external recirculation mode with two prepared SILMs and modeled results for the operation with recirculation (improved K) and without recirculation (base K).

The results under open configuration conditions (with external recirculation) revealed that the effects produced by the agitation due to the inlet and outlet streams improved the transport through the SILM. In Figure 3, the evolution of the experimental concentrations was compared to modeled values considering a standard *K* value of 0.0162 h^−1^ (which correspond to a permeability *k* value of 5.62 × 10^−4^ m/h and a *K_S_* value of 0.0081 h^−1^) obtained from a typical closed configuration test. As can be observed, the experimental values of both prepared SILMs evolved clearly faster to the equilibrium point that the modeled ones. This fact was confirmed with the assessment of the corresponding transport parameters. For instance, the *K_S_* value calculated for SILM2 was 0.0186 h^−1^, which implied a *k* value of 12.9 × 10^−4^ m/h or a *K* value of 0.0372 h^−1^ (more than double of the standard value for a closed configuration experiment). The evolution of the concentration in both compartments according to the proposed mathematical model fitted more satisfactorily when the improved *k* value was considered instead of the base *k* value derived for closed configuration tests (Figure 3). Several studies have demonstrated that agitation of the feed close to the membrane surface increases mass transfer by diminishing the concentration polarization intensity and reducing the membrane fouling [60,61,62] and further studies should be carried out to look for more effective agitation configurations to improve the transport through SILMs.

**Figure 4:**
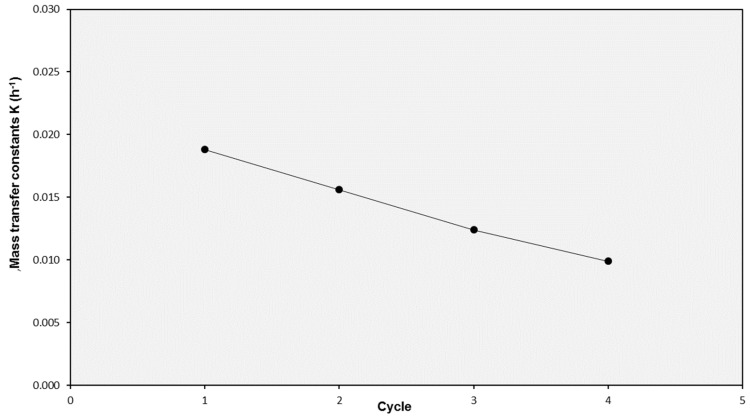
Evolution of the mass transfer coefficient K for lignosulphonate solution in SILMs used in consecutive cycles.

Finally, the influence of the number of consecutive cycles a SILM was employed was investigated. Some experiments were carried with different solutes to register the K values for the second cycle and compare them to the values obtained with the fresh SILMs. In most cases, the K values corresponding to the second cycle when compared to the first one were in the range of ±20% for all the solutes, with limited increases or decreases. However, a SILM tested with lignosulphonate solution in four consecutive cycles showed a linearly decreasing *K* value (Figure 4). Therefore, this experiment pointed to the fact that the use of these SILMs in consecutive cycles without regeneration may imply reduced performance as a consequence of fouling and/or IL loss.

**Figure 5:**
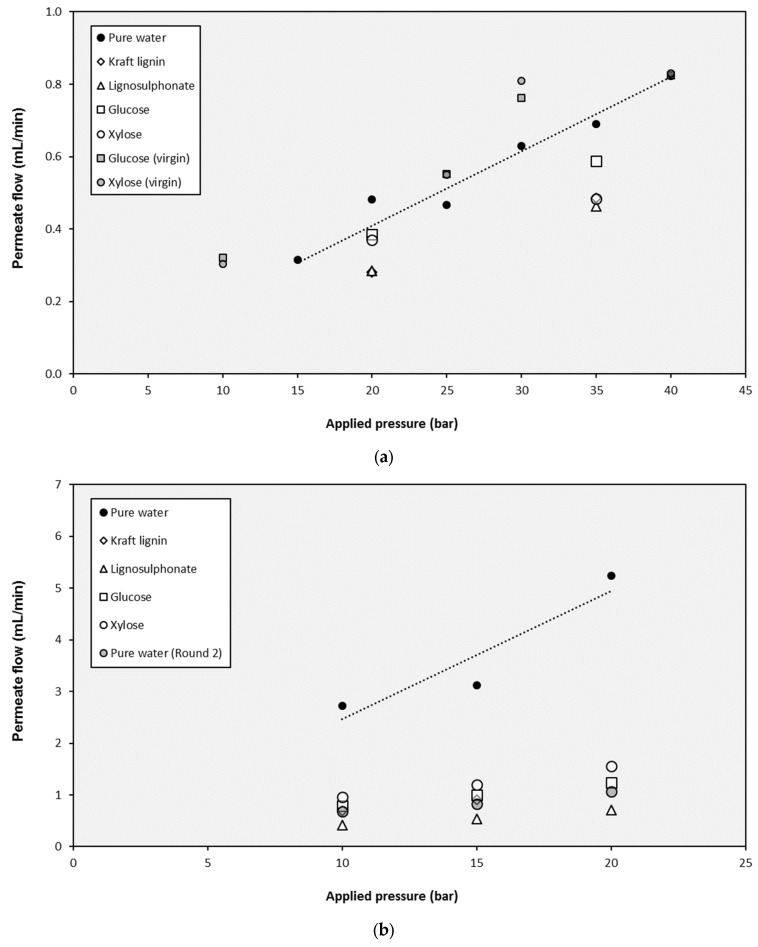

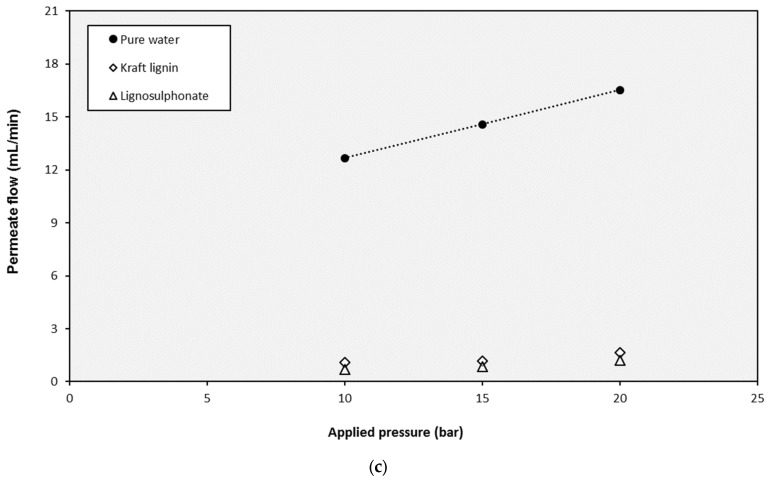
Influence of the operating pressure on the permeate production with the different solutions for (**a**) the NP010 membrane, (**b**) the UF5 membrane, and (**c**) the UF10 membrane.

The selected NF and UF membranes were used in experiments with pure water and lignin and monosaccharides solutions. The tests with pure water were useful to assess the dependence of the permeate flux (*J_P_*) on the applied pressure (Δ*P*). As shown in Figure 5, the water fluxes increased with increasing pressures, obtaining linear relationships. This linear evolution of fluxes shows that Darcy’s law is verified (Equation (11) can be applied), and the slope of these straight lines are the corresponding permeability values (*L_P_*), which are compiled in Table 2.

On the one hand, as it was expected, the highest membrane permeability corresponded to the UF10 membrane (the one with the highest molecular weight cut-off value). In the case of this membrane, the assessment of the baseline flows (*J*_0_) was also required to model its performance correctly. These values (also compiled in Table 2) were determined for the intercepts in the y-axis of the corresponding straight lines. On the other hand, the NF membrane showed the lowest permeability value, with a two-order-of-magnitude reduction when compared to UF10.

The main aspect that must be highlighted about the membrane permeabilities is the clear decrease of the values after the test with lignins (both Kraft lignin and lignosulphonates). When the NF membrane was employed, the reduction from the initial value of the test with pure water (2.34 × 10^−7^ m/s·bar) to the test with lignins was higher than 32%, and only small increases (lower than 17%) were found when the monosaccharides were tested. However, the tests with the monosaccharides using a virgin membrane without previous exposition to lignins revealed different performance: under these conditions, the permeability values of glucose and xylose (2.63 × 10^−7^ and 2.58 × 10^−7^ m/s·bar respectively) are totally comparable to the one obtained with pure water. In addition, when the UF5 membrane was investigated, the decrease of the membrane permeability with lignins was repeated. In this case, the value was reduced from 2.82 × 10^−6^ m/s·bar with pure water to 4.16 × 10^−7^ m/s·bar with lignosulphonate, which resulted in an 85% reduction, much higher than in the case of the NF membrane. Once again, some recovery of the permeability was identified when monosaccharides solutions were filtrated, but the value of the virgin membrane was not attained. An additional tested with pure water was completed after the filtration of the rest of solutions and the obtained permeability (6.38 × 10^−7^ m/s·bar) confirmed the fouling of the membranes. The flux decline of the NF and UF membrane permeability during the filtration of lignin solutions due to fouling had been previously reported by several researchers [63,64,65], so its quantification is a relevant aspect to be covered.

The characterization of the NF and UF membranes was completed with the determination of the efficiency of the selected membranes for lignins and monosaccharides removal expressed as solute rejections (Table 3). The lowest molecular weight cut-off of the NF resulted in the maximal rejection for all the solutes: from 99.5% for Kraft lignin and 98.9% for lignosulphonate to 20.0% for xylose (both values corresponded to 35 bar of applied pressure, lower values were obtained with lower applied pressure). The use of UF5 membrane implied a significant reduction of the monosaccharides rejection, with values below 11% for glucose and below 1% for xylose. Whilst, the rejection of the lignins maintained values around 99% for Kraft lignin and 95% for lignosulphonate. The UF 10 membrane was only tested for the removal of lignins (88.1% and 80.2% at 20 bar for Kraft lignin and lignosulphonate respectively), since the rejections of xylose and glucose were considered negligible.

Once both SILMs and pressure-assisted membranes were characterized, the corresponding transport parameters were employed to compare the separation performance and the selectivity coefficients of the membranes. The permeability *k* values for the four different solutes [56] and the solute passage coefficients (calculated as 100 minus the corresponding solute rejection *R*) are compiled in Table 4.

The values in Table 4 were taken as basis to calculate the separation factors *α_ij_* that define the selectivity of the membranes for the different solutes. The separation factors *α_ij_* of the SILMs were expressed as the ratio between the permeabilities of the solutes *i* and *j*:

(13) $$\alpha_{ij}=\frac{k_{i}}{k_{j}}$$

In the case of the NF and UF membranes, the separation factors *α_ij_* were expressed as the ratio between the passages of the solutes *i* and *j*:

(14) $$\alpha_{ij}=\frac{100-R_{i}}{100-R_{j}}$$

All the calculated separation factors can be observed in Table 5. The data revealed the lower selectivity of the SILM when compared to the pressure-assisted membranes. While the separation factors between lignins and monosaccharides for the SILM were lower than 3, higher values resulted for the NF and UF membranes. On the one hand, for the separation of Kraft lignin from glucose or xylose, the UF5 appeared as the optimal alternative, since separation factors higher than 100 were attained. On the other hand, when the separation of lignosulphonates from monosaccharides was desired, the NF membrane should be selected as the best option, with separation values close to 75. In any case, the SILM did not appear as a competitive alternative to compete with NF and UF membranes in this purpose.

**Figure 6:**
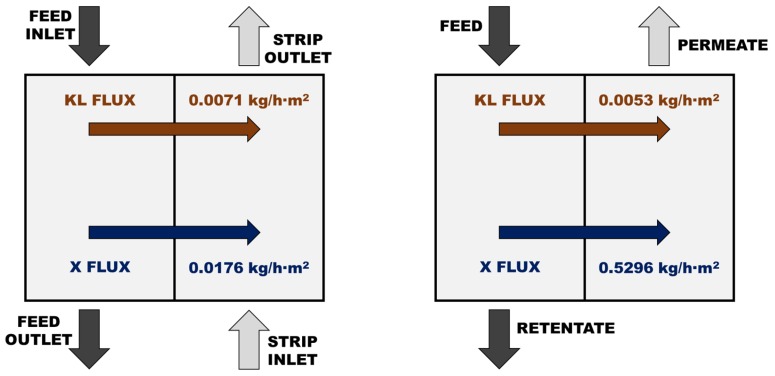
Comparison of the fluxes of Kraft lignin (KL) and xylose (X) through the SILM and the UF5 membrane.

To have a clearer idea about the lack of competitiveness of the SILM, a direct comparison of the solute transport through the SILM and the UF5 membrane was carried out. As example, the separation of a solution of 20 g/L Kraft lignin and 20 g/L of xylose was selected. In the case of the UF5 membrane, the applied pressure was 20 bar and the obtained results with both membranes are shown in Figure 6. While the fluxes of Kraft lignin can be considered comparable (just slightly lower in the case of the UF membrane), the fluxes of xylose are very different and the UF membrane allowed a more than 30 times higher transport of the monosaccharides. In order to have a better idea about the technical limitation of the SILM when compared to UF, further work was completed to assess the competitiveness limits that define the transport parameters a SILM must show to be considered competitive. On the one hand, the *k* value for xylose should be increased from 1.76 × 10^−3^ to at least 5.28 × 10^−2^ m/h to have a xylose flux through the SILM equal to the flux through the UF membrane. On the other hand, in order to compensate the selectivity of the UF membrane, the *k* value for xylose of the SILM should achieve 7.28 × 10^−2^ m/h, or, alternatively, the *k* value for Kraft lignin of the SILM should be reduced below 1.72 × 10^−5^ m/h. These figures confirmed the better performance of the UF membrane for this separation task. Consequently, the need of important improvements in the design of SILMs must be highlighted in order to be able to compete with NF and UF membranes for lignin separation [66,67,68,69,70,71,72].

## 4. Conclusions

The SILM formed with [BMIM][DBP] as IL and PTFE as membrane support demonstrated selective transport of two different types of technical lignins (Kraft lignin and lignosulphonate) and monosaccharides (xylose and glucose) in aqueous solutions. The SILM maintained its performance under different conditions during the preparation procedure and, in addition, the selection of an open configuration in the system allowed the agitation of the solution in contact with the SILM and improved the transport of the solutes. However, the stability of the SILMs was not adequate to use them in consecutive cycles and may be problematic to use them for long time periods without regeneration. Nevertheless, the investigated SILM was not competitive when compared to pressure-assisted nanofiltration (NF) and ultrafiltration (UF) membranes, although these NF and UF ones suffer severe fouling, which has been quantified in this study as the flux decline during the filtration of lignin solutions. The use of the NF membrane was the optimal option for the separation of lignosulphonates from monosaccharides (separation factors above 25 times higher than the SILM), while the UF5 membrane appeared as the best solution to separate Kraft lignin and monosaccharides (separation factors above 30 times higher than the SILM).

## Figures and Tables

**Table 1:** Characteristics of the NF and UF membranes used in this work.

Characteristics	NF Membrane	UF Membranes	
Manufacturer	Microdyn-Nadir	Trisep	Trisep
Model	NP010	UF5	UF10
Material	PES (effective layer) PP (support layer)	PES (effective layer) PP (support layer)	PES (effective layer) PP (support layer)
Molecular weight cut-off (Da)	1000–1200	5000	10,000
Maximum operation temperature (°C)	-	45	45
Maximum operation pressure (bar)	40	21	21
pH range	0–14	1–12	1–12
NaCl rejection (%)	10		
Na_2_SO_4_ rejection (%)	35–75		

**Table 2:** Permeability and baseline flow values of the tested membranes with the different solutions.

Permeability *L_P_* Values (m/s·bar)			
Solution	NF Membrane	UF Membranes	
	NP010	UF5	UF10
Pure water	2.34 × 10^−7^	2.82 × 10^−6^	1.06 × 10^−5^
Pure water (used membrane)		6.38 × 10^−7^	
Kraft lignin	1.59 × 10^−7^	7.25 × 10^−7^	9.69 × 10^−7^
Lignosulphonate	1.53 × 10^−7^	4.16 × 10^−7^	6.92 × 10^−7^
Glucose	1.71 × 10^−7^	9.27 × 10^−7^	
Glucose (virgin membrane)	2.63 × 10^−7^		
Xylose	1.99 × 10^−7^	7.49 × 10^−7^	
Xylose (virgin membrane)	2.58 × 10^−7^		
**Baseline Flow *J*_0_ Values (m/s)**			
Pure water			1.01 × 10^−4^
Kraft lignin			5.15 × 10^−6^
Lignosulphonate			1.65 × 10^−6^

**Table 3:** Influence of the operating pressure on the solute rejection of the NP010, UF5 and UF10 membranes.

Solute	Applied Pressure Δ*P* (bar)	Rejection (%)		
		NP010	UF5	UF10
Kraft lignin (KL)	10	-	99.0	86.9
	15	-	99.0	86.8
	20	99.0	-	88.2
	35	99.5	-	-
Lignosulphonate (LS)	10	-	95.0	74.9
	15	-	94.9	77.9
	20	98.9	94.6	80.1
	35	98.9	-	-
Glucose (G)	10	-	5.1	-
	15	-	2.0	-
	20	13.9	10.9	-
	35	40.6	-	-
Xylose (X)	10	-	0.36	-
	15	-	0.25	-
	20	14.0	0.42	-
	35	20.0	-	-

**Table 4:** Solute permeability values *k* of the SILM and solute passage percentages of the NF and UF membranes.

Solute	SILM	NF Membrane	UF Membranes	
		NP010	UF5	UF10
	*k* Value (m/h)	Solute Passage (%)		
Kraft lignin (KL)	0.71 × 10^−3^	1.0	1.0	13.2
Lignosulphonate (LS)	0.67 × 10^−3^	1.2	5.1	22.1
Glucose (G)	1.86 × 10^−3^	86.1	98.0	100.0
Xylose (X)	1.76 × 10^−3^	86.0	99.8	100.0

**Table 5:** Separation factors α_ij_ of the different solutes for the SILM and NF and UF membranes.

Solute	SILM			NF Membrane			UF Membranes					
				NP010			UF5			UF10		
	*α_i/KL_*	*α_i/LS_*	*α_i/G_*	*α_i/KL_*	*α_i/LS_*	*α_i/G_*	*α_i/KL_*	*α_i/LS_*	*α_i/G_*	*α_i/KL_*	*α_i/LS_*	*α_i/G_*
Kraft lignin (KL)	-	-	-	-	-	-	-	-	-	-	-	-
Lignosulphonate (LS)	0.94	-	-	1.2	-	-	5.3	-	-	1.7	-	-
Glucose (G)	2.63	2.80	-	88.8	74.9	-	101	19.2	-	7.6	4.5	-
Xylose (X)	2.49	2.65	0.95	88.6	74.8	1.0	103	19.6	1.0	7.6	4.5	1.0
